# Super rapid removal of copper, cadmium and lead ions from water by NTA-silica gel†

**DOI:** 10.1039/c8ra08638a

**Published:** 2019-01-02

**Authors:** Yulian Li, Junyong He, Kaisheng Zhang, Tao Liu, Yi Hu, Xifan Chen, Chengming Wang, Xingjiu Huang, Lingtao Kong, Jinhuai Liu

**Affiliations:** Nano-Materials and Environmental Detection Laboratory, Institute of Intelligent Machines, Chinese Academy of Sciences Hefei 230031 People's Republic of China kszhang@iim.ac.cn ltkong@iim.ac.cn +86-551-65592420 +86-551-65591142; Department of Chemistry, University of Science and Technology of China Hefei Anhui 230026 PR China; Cilin & CAS Environmental Science and Technology (Anhui) Inc. China; Hefei National Laboratory for Physical Sciences at the Microscale, University of Science and Technology of China Hefei Anhui 230026 PR China

## Abstract

Copper (Cu^2+^), cadmium (Cd^2+^) and lead ions (Pb^2+^) are toxic to human beings and other organisms. In this study, a silica gel material modified with nitrilotriacetic acid (NTA-silica gel) was sensibly designed and prepared *via* a simple amidation procedure for the removal of Cu^2+^, Cd^2+^ and Pb^2+^ from water. The NTA-silica gels showed rapid removal performances for the three metal ions (Pb^2+^ (<2 min), Cu^2+^ and Cd^2+^ (<20 min)) with relatively high adsorption capacities (63.5, 53.14 and 76.22 mg g^−1^ for Cu^2+^, Cd^2+^ and Pb^2+^, respectively). At the same concentration of 20 mg L^−1^, the removal efficiencies of the three metals by the adsorbent ranged from 96% to 99%. The Freundlich and Langmuir models were utilized to fit the adsorption isotherms. The adsorption kinetics for the three metal ions was pseudo-second-order kinetics. The removal performance of the NTA-silica gels increased in a wide pH range (2–9) and maintained in the presence of competitive metal ions (Na^+^, Mg^2+^, Ca^2+^ and Al^3+^) with different concentrations. In addition, the NTA-silica gels were easily regenerated (washed with 1% HNO_3_) and reused for 5 cycles with high adsorption capacity. This study indicates that the NTA-silica gel is a reusable adsorbent for the rapid, convenient, and efficient removal of Cu^2+^, Cd^2+^, and Pb^2+^ from contaminated aquatic environments.

## Abstract 2

A silica gel material modified with nitrilotriacetic acid (NTA-silica gel) was sensibly designed and prepared *via* a simple method for the super rapid removal of Cu^2+^, Cd^2+^ and Pb^2+^ from water.

## Graphical abstract

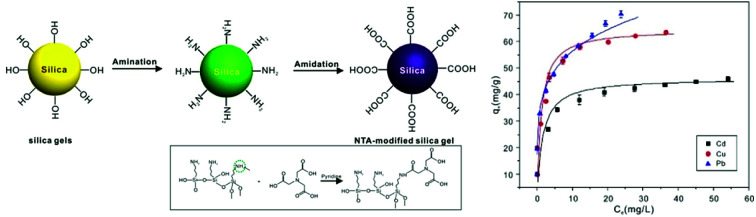

## 1. Introduction

Heavy metal ion pollution in wastewater is a significant hazard to human existence worldwide due to the non-biodegradability and high toxicity of metal ions to life. Particularly, due to the rapid globalization, the smelt industry, metal plating, electrolysis, mining operations, pigment and other industries discharge vast industrial sewage containing heavy metal ions,^1–3^ and the concentrations of these metal ions are frequently higher than the national effluent emission standards for wastewater, especially drinking water. There are various types of metal ions inside effluents including Cu^2+^, Cd^2+^, and Pb^2+^, which easily accumulate inside living organisms, thus endangering the public health. The World Health Organization has set the maximum limits of 2.0 mg L^−1^ Cu^2+^, 0.003 mg L^−1^ Cd^2+^, and 0.01 mg L^−1^ Pb^2+^ for drinking water. Therefore, the development of promising technologies to remove and reduce these heavy metal ion pollutants is urgent.

Abundant successful experimental technologies have been used to remove heavy metal ions from effluents, including chemical co-precipitation,^4^ ion exchange,^5,6^ reverse osmosis,^7^ ultrafiltration,^8^ membrane filtration,^9^ coagulation,^10^ adsorption^11,12^ and others.^13^ Among them, the adsorption technology is known as a high-efficiency method due to its effectiveness, simplicity, low cost,^14–18^ and regenerable adsorption capacity.^11,19^ At concentrations lower than 100 mg L^−1^, the removal of metal ions better accomplished by adsorption.^20^ Due to the advancement of adsorption technology, the development of new adsorbents has increased in recent years. However, to date, most of the adsorbents have poor adsorption capacities, ineffectiveness for low metal ion concentrations, slow kinetics and high costs.^21–24^

Recently, chemically modified organic polymers with functional groups such as sulfonate, amine and carboxylic have been employed to promote metal removal from the aqueous environment due to their abundant active sites for the complexation of metals.^25,26^ Nitrilotriacetic acid^27–30^ is a good complexing agent used to remove heavy metals by chelating heavy metals in aqueous solution similarly to ethylenediaminetetraacetic acid. On the other hand, although the complexation with nitrilotriacetic acid is not as strong as ethylenediaminetetraacetic acid, it has no adverse effect on aquatic creatures due to its biodegradability. However, some defects such as non-regeneration, swelling and loss of mechanical properties restrict the development of these organic materials.^31^ Thus, inorganic materials have been employed to address these issues. Silica gel, an inorganic polymer, is well-known due to its application in chromatographic columns.^32^ Its porous surface structure and large specific surface area of above 700 m^2^ g^−1^ play important roles for its interaction with metal ions. In addition, as a matrix for immobilizing different chemical functional groups, silica gel is readily accessible.^33^ It can be modified with many organic molecules by silanization methods^34^ to enhance its removal efficiency due to is superficial abundant hydroxyl groups. Among of these organic molecules, nitrilotriacetic acid (NTA)^35^ is one of the strongest chelating agents, which can be used to synthesize stable structures to arrest target metals. Furthermore, the metal binding can be reversed after chemical treatment.

In this study, we developed a new heavy metal ion adsorbent, NTA-silica gel, which was synthesized using aminated silica gel and the chelating agent NTA based on a cost-effective and easy-operation amidation reaction. The as-prepared adsorbent exhibited high efficiency for the removal of Cu^2+^, Cd^2+^, and Pb^2+^ metal ions from water. Considering the stable amide structure and chelation of NTA, the NTA-silica gel adsorbent is very suitable for the adsorption of these heavy metal ions in waste water. Subsequently, we investigated the adsorption kinetics, thermodynamic and removal capacity of NTA-silica gel through a series of adsorption experiments. The initial heavy metal ion concentration, effects of pH, and co-existing ions were explored. The heavy metal ion adsorption mechanism was examined *via* FT-IR, XPS and TG analyses. Finally, the test parameters of regeneration/recycling were also studied. The results displayed that the NTA-silica gel adsorbent exhibits excellent adsorption performances for the removal of Cu^2+^, Cd^2+^ and Pb^2+^ metal ions from water.

## 2. Materials and methods

### 2.1. Materials

Silica gel modified with amino groups (cation silica gel (19613-168B)) was commercially obtained from Aladdin Industrial Corporation (1.196 nm average particle size and 249.865 m^2^ g^−1^ special surface area), and nitrilotriacetic acid (NTA) and all other reagents were purchased from Shanghai Chemical Reagents Company. All reagents were of analytical grade and used without any further treatment. All necessary aqueous solutions were prepared using ultrapure water (18.25 MΩ cm). The stock solutions of copper, lead and cadmium for AAS (1000 mg L^−1^) were prepared by dissolving fixed quantities of Cu(NO_3_)_2_·5H_2_O, Pb(NO_3_)_2_ and Cd(NO_3_)_2_·4H_2_O in deionized water. Sodium nitrate (NaNO_3_) was added to the copper, lead and cadmium standard solutions as a buffer agent. The pH of all the solutions was adjusted using 0.1 mol L^−1^ HCl and 0.1 mol L^−1^ NaOH.

### 2.2. Synthesis of NTA-silica gel

Cation silica gel (19613-168B) (4.05 g) and NTA (3.35 g) were dispersed in 400 mL pyridine in a 1000 mL round-bottom flask. The mixture was rotated at 400 rpm in an oil bath at 90 °C with reflux system for 3 h. After it cooled to room temperature, 0.5 mol L^−1^ sodium bicarbonate was added to neutralize the unreacted NTA. Deionized water, ethanol and diethyl ether were used to rinse the particles twice, respectively. The products were dried at room temperature for 24 h, and then stored in a covered bottle before used.

### 2.3. Batch sorption experiments

Batch adsorption experiments were carried out to explore the adsorption properties and mechanism *via* a series of orthogonal experiments with the initial metal concentration, adsorption temperature, adsorption time, and solution pH values.

80 mg NTA-silica gel and 80 mL 20 mg L^−1^ Cu^2+^, Cd^2+^ and Pb^2+^ were mixed in nitrate solution with acidic conditions in 250 mL glass beaker. Then, the mixed solutions were shaken at 180 rounds per minute at room temperature for 720 min. At specific intervals, the appropriate suspension solutions were extracted with a 0.5 mL syringe and then filtered through a membrane filter. Similar conditions were applied for the adsorption kinetics experiments of Pb^2+^ except for the first extraction time, which ranged from 0.5 to 720 min.

The adsorption isotherms experiments were aimed at examining the adsorption capacities of the NTA-silica gel. The adsorption experiments were carried in 15 mL polypropylene centrifuge tubes with 10 mg NTA-silica gel and 10 mL Cu^2+^, Cd^2+^ and Pb^2+^ ions solutions in various concentrations (10–100 mg L^−1^) at pH = 5 ± 0.1 (avoiding precipitate with hydroxyl). After that, they were shaken at 180 rpm at different temperatures, specifically 25 °C, 40 °C and 50 °C, for 24 h to explore the adsorption isotherms of the three metal ions. Next, after adsorption, the solutions were filtered through a 0.22 μm pore size membrane filter following centrifugation.

In the study of the influence of pH, the same conditions as the adsorption kinetics experiment were used, where 10 mg adsorbent and 10 mL 20 mg L^−1^ metal solution were added to 15 mL polypropylene centrifuge tubes and shaken at 180 rpm at 25 °C for 24 h. The pH of the metal solution was adjusted in the range of 2.0 to 9.0 using 0.1 mol L^−1^ HCl and 0.1 mol L^−1^ NaOH.

Considering the multiformity of environmental water samples, the effect of four co-existing cations (Na^+^, Mg^2+^, Ca^2+^, and Al^3+^) on the removal of the three metal ions in wastewater was explored. To explore the effect of these four co-existing cations on removal of the three metal ions in wastewater, 10 mg adsorbent and 10 mL of 20 mg L^−1^ Cd^2+^, Cu^2+^ and Pb^2+^ ion solutions were mixed in 15 mL polypropylene flasks and three concentrations levels (0, 5, 15, 20, 30, and 80 mg L^−1^) of the co-existing cations (Na^+^, Mg^2+^, Ca^2+^, and Al^3+^) were added to the flasks with pH = 5.0 ± 0.1. The mixtures were shaken at 180 rpm in a shaker at 25 °C for 24 h, and then the suspension was separated by centrifugation and membrane filtration.

### 2.4. Analysis

A PERSEE atomic absorption spectrometer (TAS-990) (AAS) was used to test the concentration of Cu^2+^, Cd^2+^ and Pb^2+^. All the measurements were performed in triplicate and the averages of the results were prepared for data analysis. The relative error was about 0.05. The metal ion adsorption percentage, adsorption capacity and distribution coefficient (*K*_d_) were calculated as follows:^36^1
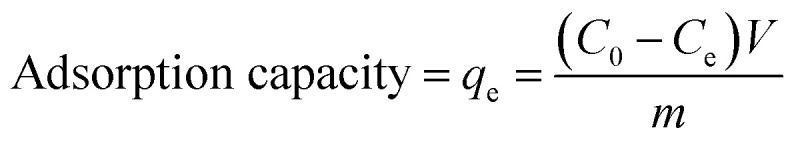
2
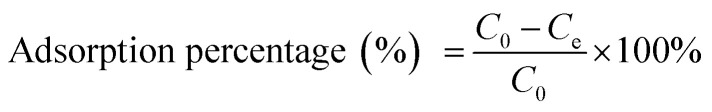
3
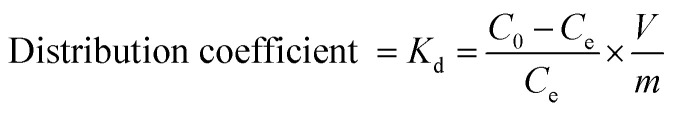
where, *C*_0_ and *C*_e_ are the initial and final equilibrium concentrations (mg L^−1^) of the metal ions in solution, respectively, *V* is the volume (L) of solution, and *m* is the mass (mg) of the adsorbent.

The three metal ions equilibrium adsorptions were analyzed based on the Langmuir^37^ and Freundlich^38^ isotherms using eqn (4) and (5), respectively.4
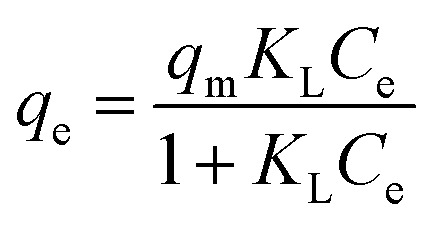
5
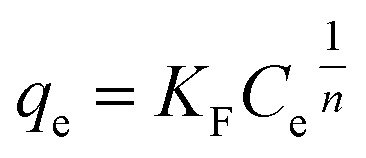
where, *K*_L_ and *K*_F_ are the Langmuir and Freundlich adsorption equilibrium constants (L mg^−1^), respectively. The Langmuir model is based on monolayer adsorption; whereas, the Freundlich model assumes the adsorption process occurs on heterogeneous surfaces.^39^

The reaction kinetics was analyzed using the pseudo-first-order and pseudo-second-order models using eqn (6) and (7).^40^6
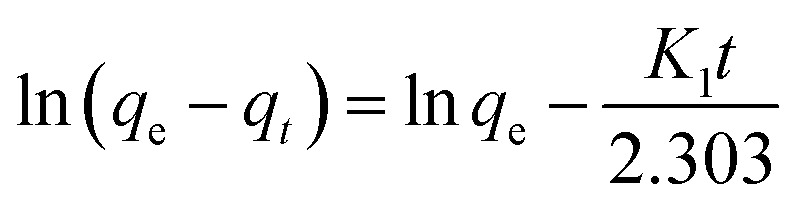
7
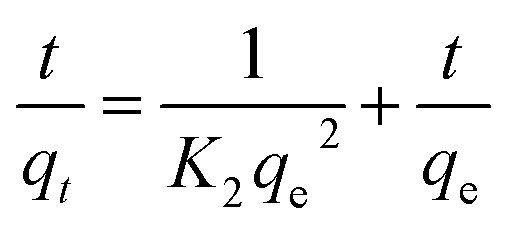
where, *q*_e_ and *q*_*t*_ are the adsorption capacity (mg g^−1^) of the C-silica gel at equilibrium and at time *t*, respectively. *K*_1_ (min^−1^) and *K*_2_ (g mg^−1^ min^−1^) are the equilibrium rate constant of pseudo-first-order adsorption and pseudo-second-order adsorption, respectively.

### 2.5. Regeneration and reuse studies

Regeneration and reuse experiments for an adsorbent are necessary for economy today's society. Thus, to investigate the reversibility of metal ion adsorption with the NTA-silica gel, 10 mL 20 mg L^−1^ Cu^2+^, Cd^2+^ and Pb^2+^ were mixed with 10 mg of NTA-silica gel in centrifuge tubes. 0.1 mol L^−1^ HCl was added and then the solutions were ultrasonically treated at room temperature for 1 h immediately following adsorption. The metal ion concentrations were determined *via* AAS. Five cyclic adsorption–desorption processes were conducted to study the regenerability of the NTA-silica gel. Reductions in the adsorption capacity of the NTA-silica gel were observed for each cycle.

### 2.6. Nanocomposite characterization

The NTA-silica gel morphological property was observed *via* field-emission scanning electron microscopy (FE-SEM, QUANTA 200 FEG, FEI Sirion). Its specific surface area, average pore diameter and pore volume were analyzed on a Brunauer–Emmett–Teller instrument (BET, Tristar II 3020M, Micromeritics American) using nitrogen adsorption with a degassing temperature of 80 °C. X-ray photoelectron spectrometry (XPS, ESCALAB 250, Thermo-VG Scientific) was carried out on an ESCA Lab MK II using non-monochromatized Mg Kα X-ray beams as the excitation source. The zeta potentials under different pH were measured on a Delsa Nano C/Z. Fourier transform infrared (FTIR) spectroscopy using potassium bromide pellets was performed on a Nexus-870 spectrophotometer to exam the functional groups of the N-silica gel composite. Thermo-gravimetric analysis (TGA, SDT Q600, TA USA) was conducted at heating rate 10 °C min^−1^ under a nitrogen flow.

## 3. Results and discussion

### 3.1. Synthesis and characterization of the NTA-silica gel

**Fig. 1:**
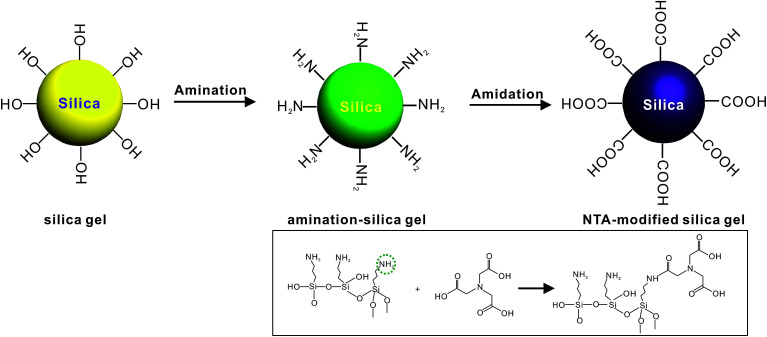
Schematic diagram of the synthesis of the NTA-silica gel.

The synthesis of the NTA-silica gel consisted of two steps, which are schematically presented in Fig. 1. First, the raw silica gel was functionalized with amino groups *via* the amination of its surface hydroxyl groups.^41^ After amino coating, NTA was grafted onto the silica gel-modified amino groups.^42^ Finally, the amino groups and carboxylic acid groups on the complexing agent were combined *via* the formation of amide bonds.^42^ Herein, the second step was performed using silica gel functionalized with amino groups (cationic silica gel (19613-168B)), which was purchased from W. R. Grace Pte. Ltd. In addition, not all the surface-bound amino groups completely reacted with NTA, although excess carboxyl groups were used in the synthesis. This can be attributed to the steric hindrance caused by the bulky organic groups of NTA.

**Fig. 2:**
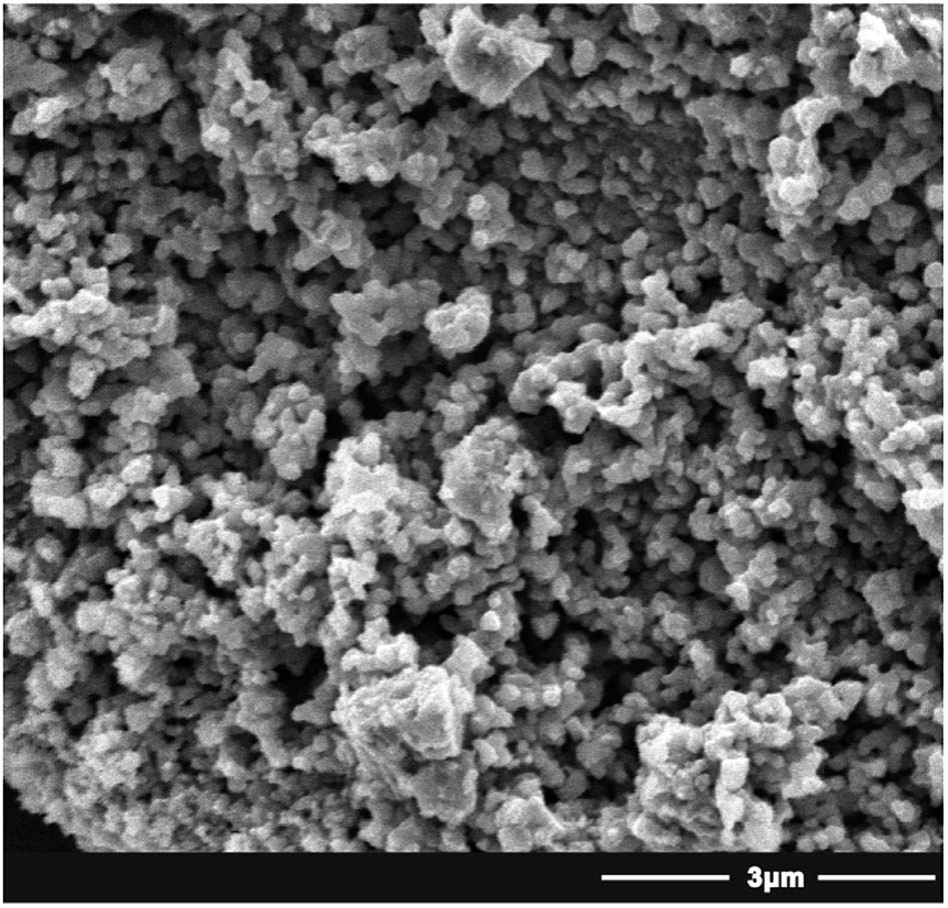
SEM micrograph of the NTA-silica gel.

**Fig. 3:**
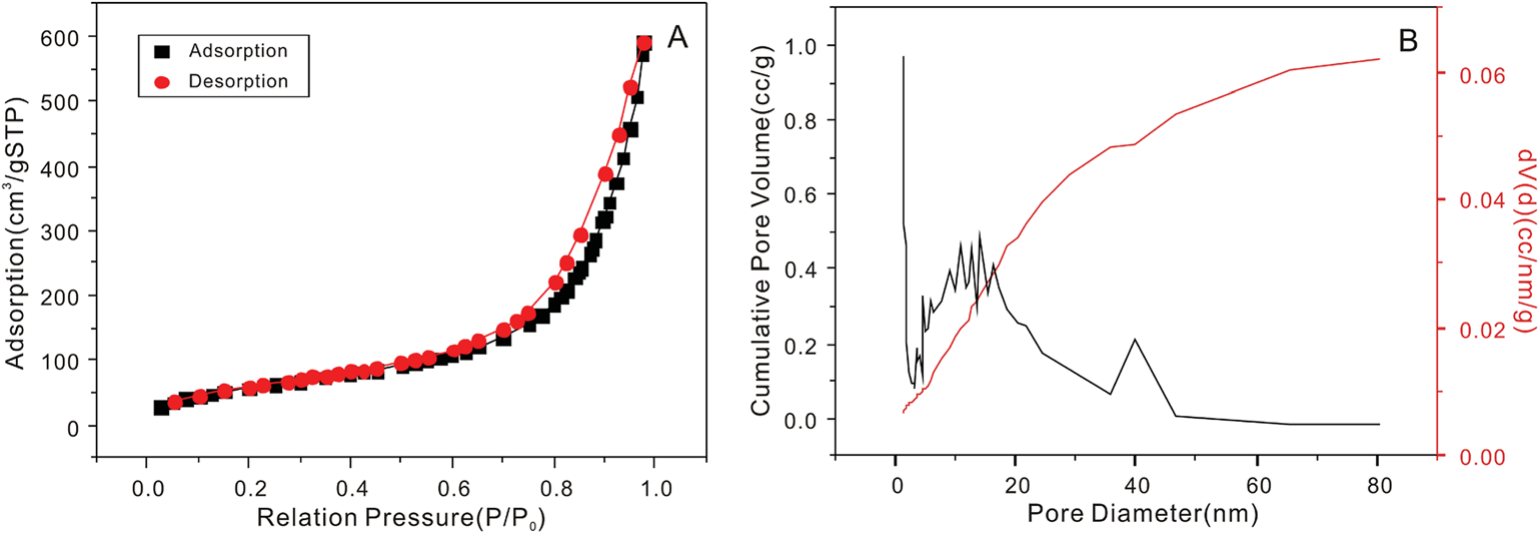
(A) Nitrogen adsorption–desorption isotherm and (B) pore-size distribution curve of the NTA-silica gel.

The SEM micrographs of the NTA-silica gel are shown in Fig. 2, which possessed a porous surface structure. The results of the surface properties of the NTA-silica gel measured by the Brunauer–Emmett–Teller (BET) method are shown in Fig. 3, which presents the N_2_ adsorption–desorption isotherms and BJH adsorption pore size distribution of the adsorbent. Both the nitrogen adsorption–desorption isotherms were type IV with a distinct hysteresis loop (*P*/*P*_0_ ≈ 1.0), indicating a mesoporous structure.^43^ The BET surface area of the NTA-silica gel was approximately 342.725 m^2^ g^−1^, which is higher than that of the aminated silica gel (249.865 m^2^ g^−1^) (see Fig. S1A in ESI†). A comparison of the BET properties of the amino-modified silica (19613-168B) and NTA-silica gel is shown in Table 1. The product presented a narrow pore size distribution with an average pore diameter of approximately 1.194 nm, which is favorable for the adsorption of pollutants. This can be attributed to the reduced pore sizes and pore blocking of the modified adsorbents. The pore size distribution was affected by the size of the attached organic group and the size of the particle. After surface modification, the number of smaller pores increased, while the number of larger pores decreased due to the formation of functional groups inside the pores.

**Table:** Comparison of the BET properties of the amino-modified silica (19613-168B) and the NTA-silica gel

Adsorbent	Diameter (nm)	Surface area (m^2^ g^−1^)	Pore volume (cm^−3^ g^−1^)
Amino-modified silica gel	1.196	249.865	0.862
NTA-silica gel	1.194	342.725	0.961

**Fig. 4:**
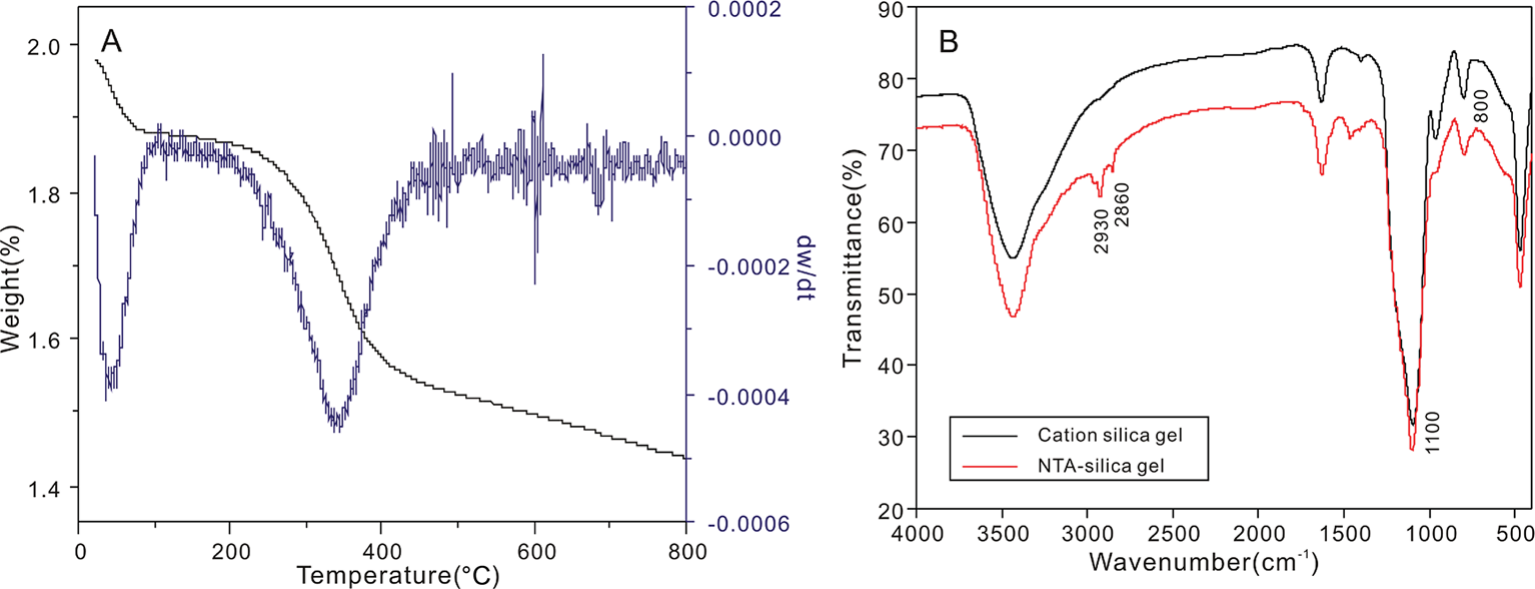
(A) Thermogravimetric analysis (TGA) of the NTA-silica gel and (B) FTIR spectra of the cationic silica gel and NTA-silica gel.

The TGA curves of the carboxyl-modified silica gel (Fig. 4A) showed two weight loss steps at about 50 °C and 240 °C, which can be attributed to the loss of adsorbed H_2_O and the decomposition of NTA, respectively. The FTIR spectrum of the NTA-silica gel is shown in Fig. 4B. The characteristic peaks at 1100 (asymmetric) and 800 (symmetric) cm^−1^ correspond to Si–O–C.^44^ The peaks at 2930 and 2860 cm^−1^ are attributed to the stretching vibration (asymmetric and symmetric) of methylene, which indirectly confirmed that NTA was bonded on the surface of the cationic silica gel.

### 3.2. Adsorption kinetics of Cu^2+^, Cd^2+^ and Pb^2+^

**Fig. 5:**
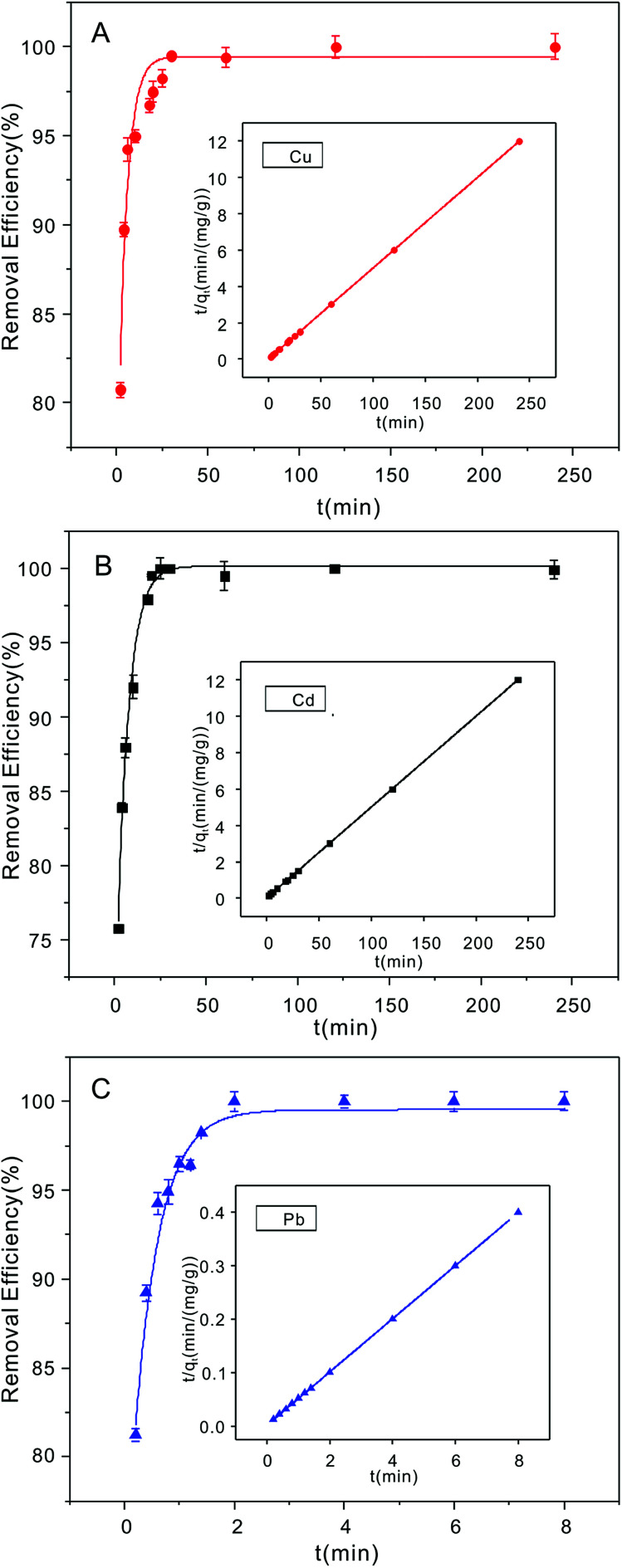
Adsorption uptake time and adsorption kinetics fitted by the pseudo-second model order with (A) Cu^2+^, (B) Cd^2+^ and (C) Pb^2+^ on the NTA-silica gel in solution at pH 5.0 with the same initial concentration (adsorbent dose: 1 g L^−1^, initial concentration: 20 mg L^−1^).

The time-dependent metal ion removal (20 mg L^−1^ initial concentration) with the NTA-silica gel (1 g L^−1^) showed the extraordinarily rapid adsorption of Cu^2+^ and Cd^2+^ in the first twenty minutes (Fig. 5A and B, respectively). Furthermore, the adsorption of Pb^2+^ was the best. In just two minutes (Fig. 5C), it reached adsorption equilibrium, which is much faster than reported for the adsorption materials shown in Table 2. Also, all the removal efficiencies were greater than 98%. The plentiful carboxyl groups on NTA play an important role for the access of these three metal ions with comparatively rapid metal ion sorption kinetics.

**Table:** Comparison of the adsorption capacities of the different adsorbents for the removal of heavy metal ions

Adsorbent sample	Adsorption capacities (mg g^−1^)			Equilibrium time (min)			Reference
	Cu^2+^	Cd^2+^	Pb^2+^	Cu^2+^	Cd^2+^	Pb^2+^	
Resacetophenone-loaded silica gel	12.31	7.01	14.55	>25	>25	>25	45
DHAQ-loaded silica gel		7.89	15.75		>30	>30	46
GASG	15.38	6.09	12.63	30	30	30	47
Amino-functionalized silica gel		49.52	143.29		100	100	48
Thiol-modified silica gel		10.43	9.41		200	200	49
Nano-TiO_2_ immobilized on silica gel			3.16			25.5	50
l-Alanine immobilized on carbon			40			3	51
BP-2/WP-2	116/130			200			52
NH_2_/MCM-41/NTAA			147			60	53
NTAA-LCM		143.4	303.5		5	5	54
Zincon–Si–MNPs			21.5			2	55
NTA-silica gel	63.5	53.14	76.22	20	20	2	This paper

The pseudo-first-order and pseudo-second-order kinetic models^56^ were employed to quantify the adsorption efficiency of Cu^2+^, Cd^2+^ and Pb^2+^ on the NTA-silica gel at a specific time. The adsorption kinetics fitted by pseudo-second-order models of Cu^2+^, Cd^2+^ and Pb^2+^ were studied based on the pseudo-second-order rate (*R*^2^ > 98%) (Table 3), which was better than the pseudo-first-order model (*R*^2^ < 93%) (see Fig. S2A–C in the ESI†).

Comparison of the pseudo-first-order and pseudo-second-order model parameters of the kinetics models of Cu^2+^, Cd^2+^ and Pb^2+^ for fitting of the experimental results

**Table:** 

Heavy metal ion	*C*_0_ (mg L^−1^)	*q*_e(exp)_ (mg g^−1^)	*k*_1_ (min^−1^)	*q*_e(cal)_ (mg g^−1^)	*R* ^2^
Pseudo-first-order model					
Cu^2+^	20	20	0.339	3.815	0.892
Cd^2+^	20	20	0.105	3.438	0.670
Pb^2+^	20	20	3.936	4.344	0.924

**Table:** 

Heavy metal ion	*C*_0_ (mg L^−1^)	*q*_e(exp)_ (mg g^−1^)	*k*_2_ (g mg^−1^ min^−1^)	*q*_e(cal)_ (mg g^−1^)	*R* ^2^
Pseudo-second-order model					
Cu^2+^	20	20	0.246	20.01	0.998
Cd^2+^	20	20	0.059	20.04	0.999
Pb^2+^	20	20	3.733	20	0.999

### 3.3. Adsorption isotherms of Cu^2+^, Cd^2+^ and Pb^2+^

**Fig. 6:**
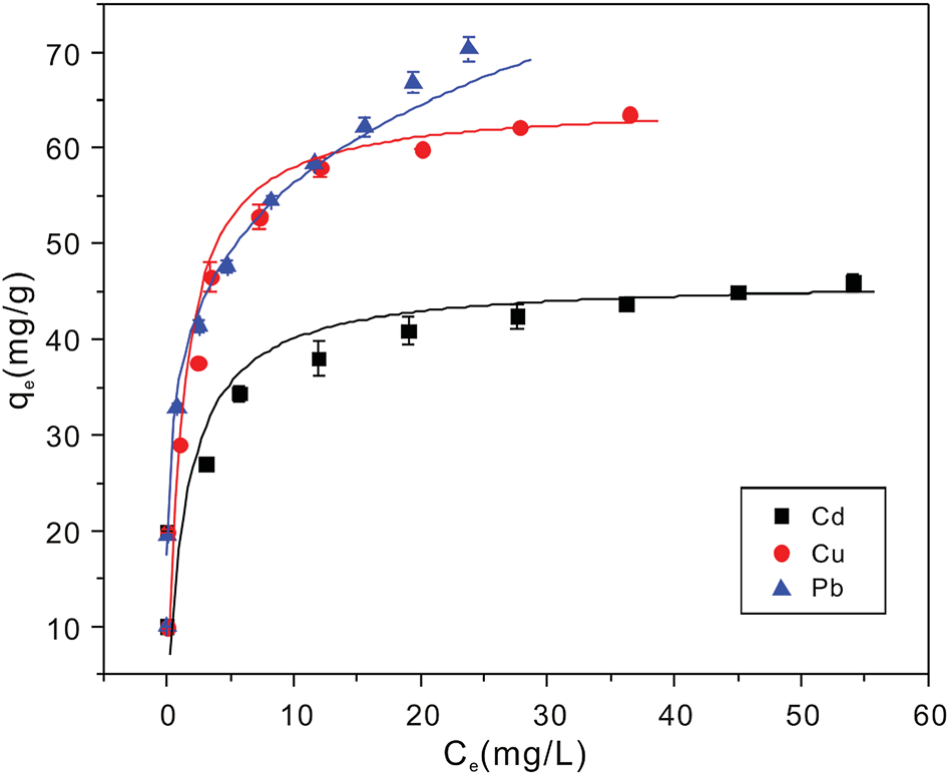
Cu^2+^, Cd^2+^ and Pb^2+^ ion adsorption isotherms on NTA-silica gel at 298 K (adsorbent dose: 1 g L^−1^, pH: 5.0).

**Fig. 7:**
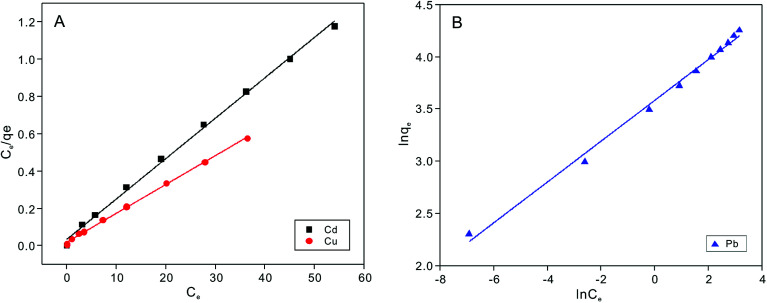
Langmuir adsorption models for fitting of (A) Cu^2+^ and Cd^2+^ and Freundlich adsorption models for fitting of (B) Pb^2+^ on the NTA-silica gel at 298 K.

Adsorption isotherms experiments were conducted to investigate the adsorption performance and the adsorption mechanism. The adsorption isotherms of Cu^2+^, Cd^2+^ and Pb^2+^ on the NTA-silica gel at 298 K, 313 K and 323 K were acquired at pH = 5.0, as shown in Fig. 6 and Fig. S3 (see ESI†). The experimental data were analyzed with the Langmuir and Freundlich adsorption models, and the relative parameters for the Langmuir and Freundlich models were calculated and displayed in Table 4, Fig. 7, S4, S5 and S6† (see ESI†). The results demonstrated that both Cu^2+^ and Cd^2+^ fitted the Langmuir adsorption model with a higher correlation coefficient (*R*^2^ > 0.99) than the Freundlich adsorption model, implying that the adsorption process occurred on a monomolecular layer. Whereas, the adsorption of Pb^2+^ was better fitted to the Freundlich adsorption model with a greater correlation coefficient (*R*^2^ = 0.971), which suggests that the adsorption process occurred on a heterogeneous surface.^57–59^

**Table:** Parameters for the Langmuir and Freundlich models of Cu^2+^, Cd^2+^ and Pb^2+^ adsorption on the NTA-silica gel at 298 K

Heavy metal ion	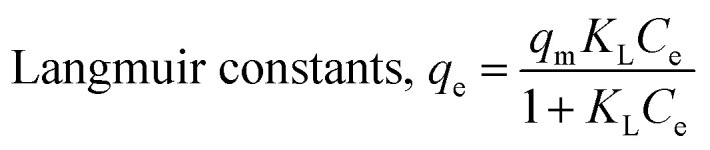				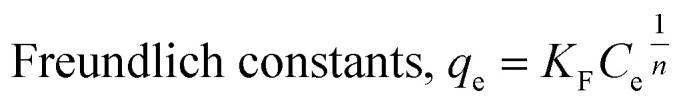		
	*q*_m(cal)_ (mg g^−1^)	*q*_m(exp)_ (mg g^−1^)	*K*_L_ (L mg^−1^)	*R* ^2^	*K*_F_ (mg^1−*n*^ L^*n*^ g^−1^)	*n*	*R* ^2^
Cu^2+^	64.27	63.51	0.909	0.998	25.55	3.40	0.922
Cd^2+^	45.25	53.14	0.781	0.996	26.64	7.04	0.988
Pb^2+^	76.05	76.22	0.573	0.962	34.79	5.12	0.971

According to the Langmuir equation, the maximum uptake capacities of Cu^2+^, Cd^2+^ and Pb^2+^ reached 63.51, 53.14 and 76.22 mg g^−1^, respectively, when the initial concentration was 100 mg L^−1^, which are much higher than that of many of the adsorption materials shown in Table 2. Moreover, the NTA-silica gel has superior affinity with Cu^2+^ Cd^2+^ and Pb^2+^ than the amino-modified silica gel (see Fig. S8 in the ESI†).

### 3.4. Adsorption thermodynamic parameter analysis

The heavy metal ion adsorption on NTA-silica gel was determined to be either an endothermic or exothermic process using the thermodynamic parameters (Δ*H*°, Δ*S*° and Δ*G*°), which were obtained from the adsorption isotherms. Also, these parameters are used to define whether the process is spontaneous or non-spontaneous. The standard free energy change (Δ*G*°) can be calculated from eqn (8) as follows:^60^

8 Δ*G*° = −*RT* ln *K*°

where, *R* is the gas constant (8.314 J (mol^−1^ K^−1^)), *T* is temperature in Kelvin, and *K*° is the adsorption equilibrium constant. ln *K*° was obtained by plotting figure of ln *K*_d_*versus C*_e_, and when the value of *C*_e_ was equal to zero, the value of the *Y*-axis was the value of ln *K*° (see Fig. S7 in the ESI†).^61,62^

The enthalpy (Δ*H*°) and the entropy change (Δ*S*°) are related to slope and intercept, respectively, in the linear plot of ln *K*° *versus* 1/*T* for heavy metal ions on NTA-silica gel according to eqn (9) as follows:^63^9
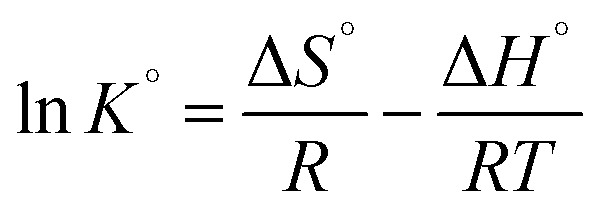


Accordingly, the linear plots of ln *K*° *versus* 1/*T* for the adsorption of heavy metal ions on the NTA-silica gel at 298, 313, and 323 K were plotted (see Fig. S7 in the ESI†). The values of Δ*S*° and Δ*H*° were calculated using the intercepts and slopes in the fitted lines, respectively. The values of the thermodynamic parameters (*i.e.*, ln *K*°, Δ*G*°, Δ*H*°, and Δ*S*°) describe the detailed processes that occurred on the NTA-silica gel. The thermodynamic parameters for the heavy metal ion adsorption are listed in Table 5. The positive Δ*H*° values illustrate that the adsorption of Cu^2+^ and Pb^2+^ on the NTA-silica gel is an endothermic process and adsorption of Cd^2+^ is an exothermic process. Additionally, the negative Δ*G*° values indicate that the adsorption of Cu^2+^, Cd^2+^ and Pb^2+^ on the NTA-silica gel is a spontaneous process.

**Table:** The thermodynamic parameters for the adsorption of Cu^2+^, Cd^2+^ and Pb^2+^ on the NTA-silica gel

Heavy metal ion	*T* (K)	Δ*H* (kJ mol^−1^)	Δ*S* (J mol^−1^ K^−1^)	Δ*G* (kJ mol^−1^)
Cu^2+^	298	42.67	173.38	−9.06
	313			−11.43
	323			−13.43
Cd^2+^	298	−26.09	−43.77	−12.99
	313			−12.57
	323			−11.85
Pb^2+^	298	24.05	121.22	−12.07
	313			−13.70
	323			−13.80

### 3.5. The effect of pH on Cu^2+^, Cd^2+^ and Pb^2+^

**Fig. 8:**
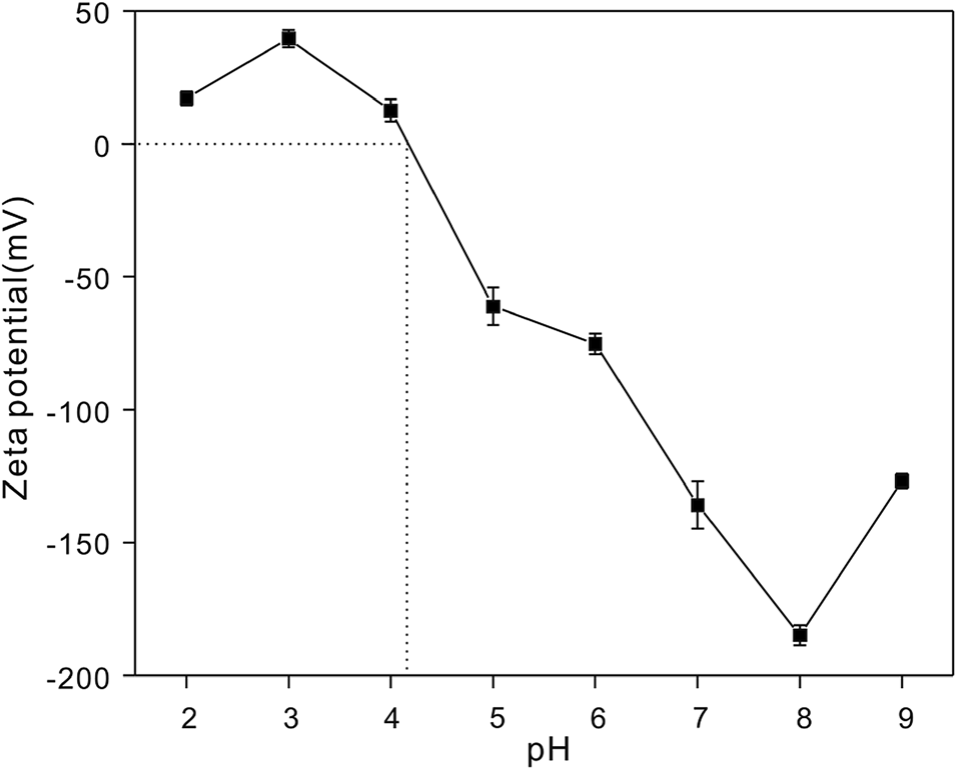
Zeta potential of the NTA-silica gel as a function of pH.

It is important to explore the effect of various H^+^ concentrations on heavy metal ion adsorption.^64^ The zeta potentials of the NTA-silica gel were determined at different pH values ranging from 2.0 to 9.0, as shown in Fig. 8. The zeta potentials of the NTA-silica gel persistently decreased with an increase in pH value. The isoelectric point determined for the NTA-silica gel existed at about pH = 4.1. Potentially, due to the release of surface protons, the surface charges of the modified silica gels changed apparently from positive to negative with an increase in pH. This process facilitated the adsorption of the metal ions from aqueous solution and was identified with the effect of pH on the adsorption of the three metal ions. The surface charge of the NTA-modified silica gel was negative at pH > 4.1, which indicated that the electrostatic interactions between the surface negative charge of the NTA-modified silica gel and surface positive charge of the heavy metals played a significant role when the pH was greater than 4.1. Electrostatic interaction is a physical adsorption process, which is faster than chemical adsorption. Furthermore, the adsorption basically included three processes: particle external diffusion (from the aqueous solution to the surface of the material), particle internal diffusion (from the material surface to the pore inside) and adsorption reaction (to the functional groups within the pores). The first two processes were slow and the last one was fast, and the adsorption rate mainly depended on the previous two processes. Also, the first two adsorption processes most likely relied on electrostatic interactions in our study. Therefore, the rapid removal of lead, copper and cadmium occurred.

**Fig. 9:**
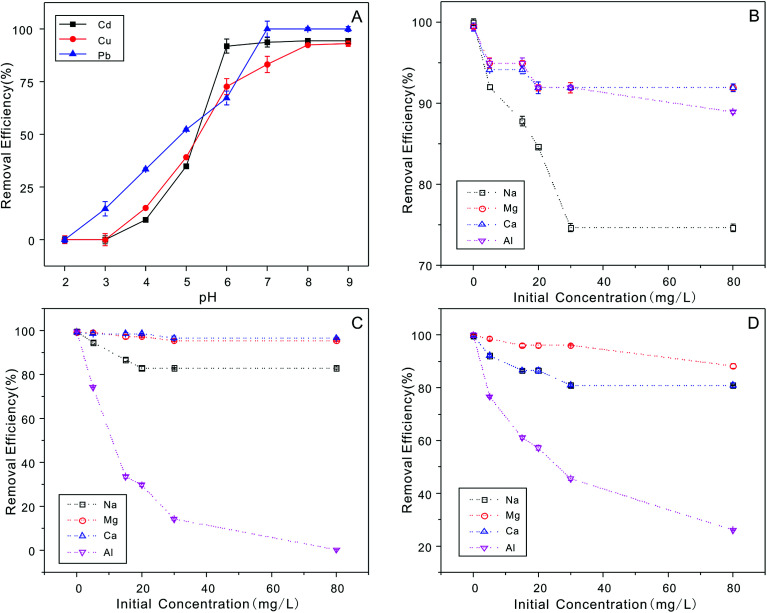
(A) Adsorption of Cu^2+^, Cd^2+^and Pb^2+^on the NTA-silica gel as a function of pH = 2.0–9.0, characterized by the removal efficiency. The effect of co-existing ions (Na^+^, Mg^2+^, Ca^2+^ and Al^3+^) on the removal of (B) Cu^2+^, (C) Cd^2+^ and (D) Pb^2+^ (adsorbent dose: 1 g L^−1^, pH: 5, 20 mg L^−1^).

The experiment to study the effect of solution pH was conducted at different pH ranging from 2.0 to 9.0 with 20 mg L^−1^ initial concentration, as shown in Fig. 9A. The removal efficiency increased gradually between pH 2.0 to 6.0 for Cu^2+^, Pb^2+^ and Cd^2+^. The maximum removal of metal ions was achieved when pH = 6.0, 7.0, and 8.0, respectively. Usually, the ionization degree, morphology and surface charge of nanomaterials are entirely different at different pH values. The electrostatic repulsion between the surface of the adsorbent and metal ions is reduced due to the loss of positive surface charge; therefore, the removal efficiency increased gradually. The removal efficiency stabilized at around 6.0 to 9.0 for Cu^2+^, Pb^2+^ and Cd^2+^ ions. This can be attributed to the limitations of the surface binding chelate and loading stability. Also, metal ions exist in various forms at different pH. When the pH > 7.0, they easily form hydroxide precipitates (*e.g.* M(OH)^+^, M(OH)_2_, and M(OH)_3_^−^). However, M(OH)_3_^−^ is difficult to be adsorbed onto the negatively charged surface of the absorbent due to electrostatic repulsion. Thus, the concentrations of the Cu^2+^, Cd^2+^ and Pb^2+^ ions dramatically decrease at pH > 8.0 because M(OH)^+^ was adsorbed onto the negatively charged surface of the absorbent, and M(OH)_3_^−^ resulted in the precipitation of M(OH)_2_ at high pH values.

### 3.6. The effect of co-existing ions

The effects of four co-existing ions (Na^+^, Mg^2+^, Ca^2+^ and Al^3+^) on the adsorption of the heavy metal ions were investigated, and the results are shown in Fig. 9B–D. The results demonstrated that these co-existing ions had little effect on the removal of Cu^2+^, and Cd^2+^ ions, and the removal efficiency remained above 80%. Nevertheless, Al^3+^ had a significant influence on the adsorption of Pb^2+^ ions. This is due to the electrostatic attractions between more electron vacant Al^3+^ and lone-pair electrons of the adsorbent. When the pH was varied from 2.0 to 9.0, it was noticed that the separation of the functional groups from the surface of the adsorbents increased at a higher pH. In summary, more functional groups could compete with metal ions on the active adsorption sites, which resulted in a decrease in the adsorption capacities for Cu^2+^, Cd^2+^ and Pb^2+^. These results are consistent with the effects of pH discussed above.

### 3.7. Removal mechanism

It was speculated that various principles including physical and chemical adsorption were involved in the metal removal. Based on the carboxylic, amine and hydroxyl functional groups on the surface of the silica gel, their interactions can be shown as follows:

10 M^2+^ + 2(–SiOHX) → (–SiOHX)_2_M + 2H^+^

where, (–SiOHX) represents the surface-modified functional group of the NTA-silica gel, X represents NTA, and M^2+^ and H^+^ are Pb^2+^, Cu^2+^ or Cd^2+^ ions and the hydrogen ions, respectively.

NTA exists as various species as H_*n*_NTA^*n*−3^ according to the thermodynamic data analysis (*n* ranges from 0 to 4). According to the experimental data, it is suggested that the adsorption removal by the carboxyl-modified silica gels can be shown as follows:

11 M^2+^_(aq)_ + [SiO–H–H_*i*_NTA]^*i*−2^_(aq)_ ↔ [SiO–M–H_*i*_NTA]^*i*−1^_(s)_ + H^+^_(aq)_

where, [SiO–H–H_*i*_NTA]^*i*−2^ represents the speciation of NTA-silica gel, M^2+^ is the divalent metal ion, and *i* (ranges from 0 to 3) is the amount of H^+^ bonded with NTA.

Eqn (8) suggests that the coulombic forces between the positive charge on the surface of the heavy metal ions and the negative charge of the adsorbent NTA-silica gel play an important role in the adsorption processes. The existence of oxygen-containing functional groups on the surface of the adsorbent, such as hydroxyl, carboxylic and carbonyl, allowed electrostatic interactions to occur between these electron-donating groups and the electron-accepting heavy metal ions, namely Cu^2+^, Cd^2+^ and Pb^2+^ ions. At an acidic pH range, the ion exchange mechanism of the three heavy metal ions possibly had a synergistic effect for their adsorption, and the protonated functional groups of the NTA-modified silica gel could facilitate the reversible ion exchange process.

The three superficial functional groups, hydroxyl, carboxylic, and amine groups, of the adsorbent donated their lone pairs of delocalized p-electrons to the surface of the metal ions to form surface oxide compounds, [SiO-M-H_*i*_NTA]^*i*−1^. Eqn (8) also demonstrates that the adsorption progress shifted from M^2+^ to produce more oxygenated metal complexes [SiO–M–H_*i*_NTA]^*i*−1^ on the adsorbent surface for a higher removal efficiency. For equilibrium, the pH decreased followed the adsorption of metal ions by the hydrophilic adsorbent.

**Fig. 10:**
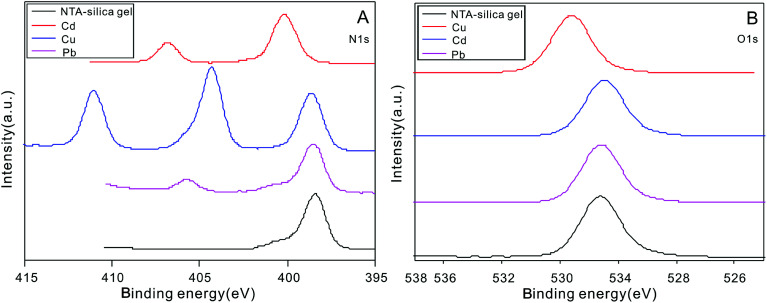
(A) XPS N1s spectra of the NTA-silica gel before and after the adsorption of Cu^2+^, Cd^2+^ and Pb^2+^. (B) XPS O1s spectra of the NTA-silica gel before and after the adsorption of Cu^2+^, Cd^2+^ and Pb^2+^.

The XPS spectrum of the NTA-silica gel was also considered to be significant in analyzing the adsorption mechanism. The XPS spectra of the NTA-silica gel before and after adsorption are shown in Fig. 10 and S9,† respectively. As revealed in Fig. S9,† the binding energy values of Cu_2p1/2_ and Cu_2p3/2_ at 934.8 and 954.8 eV and the binding energy value of Cd_3d5_ and Cd_3d2_ at 404.3 and 411.05 eV indicated that Cu^2+^ and Cd^2+^ were bound to the NTA-silica gel after adsorption, respectively. Similarly, the newly observed binding energy value of Pb_4f7/2_ and Pb_4f7/5_ at 137.55 and 142.4 eV, respectively, indicated that Pb^2+^ was bound to the adsorbents. Fig. 10A shows that the N 1s peak for the NTA-silica gel-M^2+^ assigned to the C–N group was shifted to higher energies, indicating a reduced electron density of N atoms, which is after adsorption.^65^ The lone-pair electrons of the sp^2^-bonded N atoms provided coordination sites for M^2+^ adsorption. It was speculated that the N atoms are the sites for coordination with M^2+^. In Fig. 10B, the O 1s binding energy value of M^2+^ on the NTA-silica gel shifted slightly after adsorption, indicating there were also interactions between M^2+^ and O. It has been inferred that the unoccupied orbital of M^2+^ coordinates with the lone-pair electrons between the N and O atoms.^66,67^

The FT-IR of spectra of the NTA-silica gel before and after Cu^2+^, Cd^2+^ and Pb^2+^ adsorption (see Fig. S10 in ESI†) demonstrated that the peak for C–N showed an evident red-shift, and that for O=C–NH was obviously weakened and N–H/H_2_O widened, indicating the individual amino and carboxyl groups were helpful for adsorption. These results are consistent with the with XPS spectra.

### 3.8. Desorption and regeneration

**Fig. 11:**
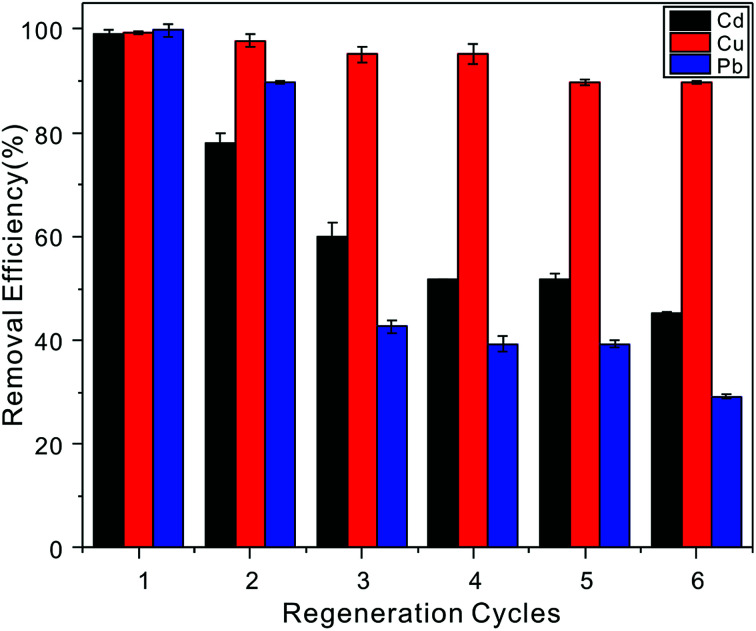
Cu^2+^, Cd^2+^ and Pb^2+^ (20 mg L^−1^ initial concentration, pH = 5.0) removal efficiency on the NTA-silica gel after six regeneration cycles.

Five continuous adsorption–desorption cycles were conducted to demonstrate the regenerability and reusability of the NTA-silica gel, and the recovery of Cd^2+^ adsorbed on the adsorbent was investigated by washing with 1% HNO_3_. The removal efficiency results are shown in Fig. 11. When the initial metal ion concentration was 20 mg L^−1^, after five cycles, it was found that a large proportion of the adsorbed Cu^2+^ (>85%) could be recovered, indicating the easy regeneration of the Cd-NTA-silica gel. However, the regeneration results for Cd^2+^ and Pb^2+^ were not good as Cu^2+^, and their removal efficiencies were lower at around 50%.

## 4. Conclusion

A novel adsorbent NTA-silica gel was developed for the removal of the heavy metal ions Cu^2+^ Pb^2+^ and Cd^2+^ from water, which showed excellence adsorption performances. The Freundlich and Langmuir adsorption models were utilized to explain the adsorption isotherms for Cu^2+^, Cd^2+^ and Pb^2+^. The adsorption kinetics of Cu^2+^, Cd^2+^ and Pb^2+^ followed the pseudo-second-order model. The results showed that the prepared adsorbent displayed very quick removal performances for the three metal ions (Pb^2+^ (<2 min), Cu^2+^ and Cd^2+^ (<20 min)), the removal efficiencies of the three metals ranged from 96% to 99%, and the adsorption capabilities approach 63.5, 53.14 and 76.22 mg g^−1^ for Cu^2+^, Cd^2+^ and Pb^2+^, respectively. Furthermore, the heavy metal ion removal mechanism was investigated *via* FTIR and XPS, which proved that the coordination between M^2+^ and the N and O atoms in the adsorbent played an important role. Moreover, the very good reusability of the NTA-silica gel demonstrates that the obtained adsorbent has great potential in environmental pollution management.

## Conflicts of interest

There are no conflicts to declare.

## Supplementary Material

RA-009-C8RA08638A-s001
