# Supplementary material for: Super rapid removal of copper, cadmium and lead ions from water by NTA-silica gel
Source: RSC Adv. 2019 Jan 2;9(1):397–407. doi: 10.1039/c8ra08638a (PMC9059273; doi:10.1039/c8ra08638a)
Supplement: RA-009-C8RA08638A-s001 [file RA-009-C8RA08638A-s001.pdf]

Electronic Supplementary Material (ESI) for RSC Advances.
This journal is © The Royal Society of Chemistry 2018

Supplementary information

Super rapid removing copper, cadmium and lead ions from water by NTA-silica gel

Yulian Li[a, b], Junyong He[a, b], Kaisheng Zhang[a, *], Tao Liu[a, b], Yi Hu[a, b], Xifan Chen [c], Chengming Wang [d], Xingjiu Huang[a], Lingtao Kong[a, *], and Jinhuai Liu[a]

[a] Nano-Materials and Environmental Detection Laboratory, Institute of Intelligent Machines, Chinese Academy of Sciences, Hefei 230031, People's Republic of China.

[b] Department of Chemistry, University of Science and Technology of China, Hefei, Anhui 230026, PR China.

[c] Cilin & CAS Environmental Science and Technology (Anhui) Inc.

[d] Hefei National Laboratory for Physical Sciences at the Microscale, University of Science and Technology of China, Hefei, Anhui 230026, PR China

Corresponding Author

*E-mail address: kszhang@iim.ac.cn (K. Zhang); ltkong@iim.ac.cn (L. Kong), Fax: +86-551-65592420; Tel: +86-551-65591142.

S1. The BET of non-NTA-modified silica gel

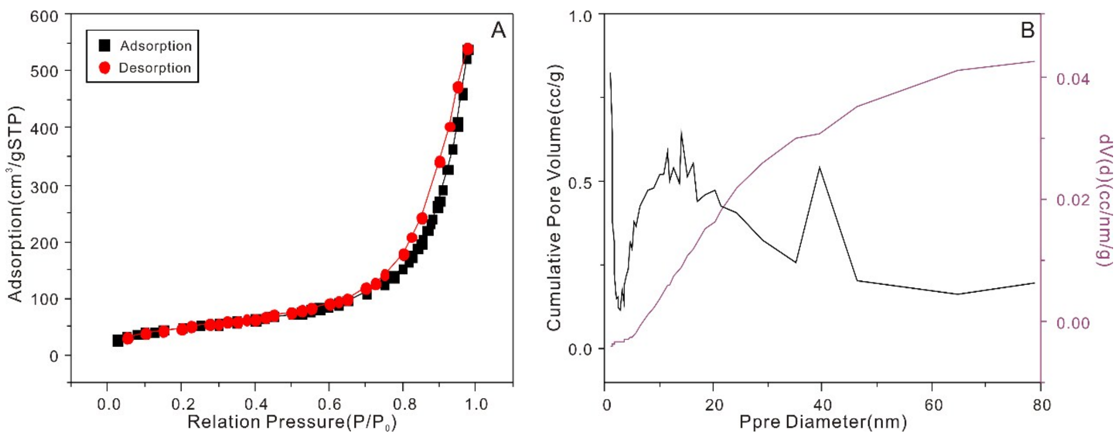

Fig. S1. (A)Nitrogen adsorption–desorption isotherm, and (B) the pore-size distribution curve of the non-NTA-modified silica gel.

S2. The pseudo-first-order kinetic plots for the adsorption of Cu^{2+}, Cd^{2+} and Pb^{2+}

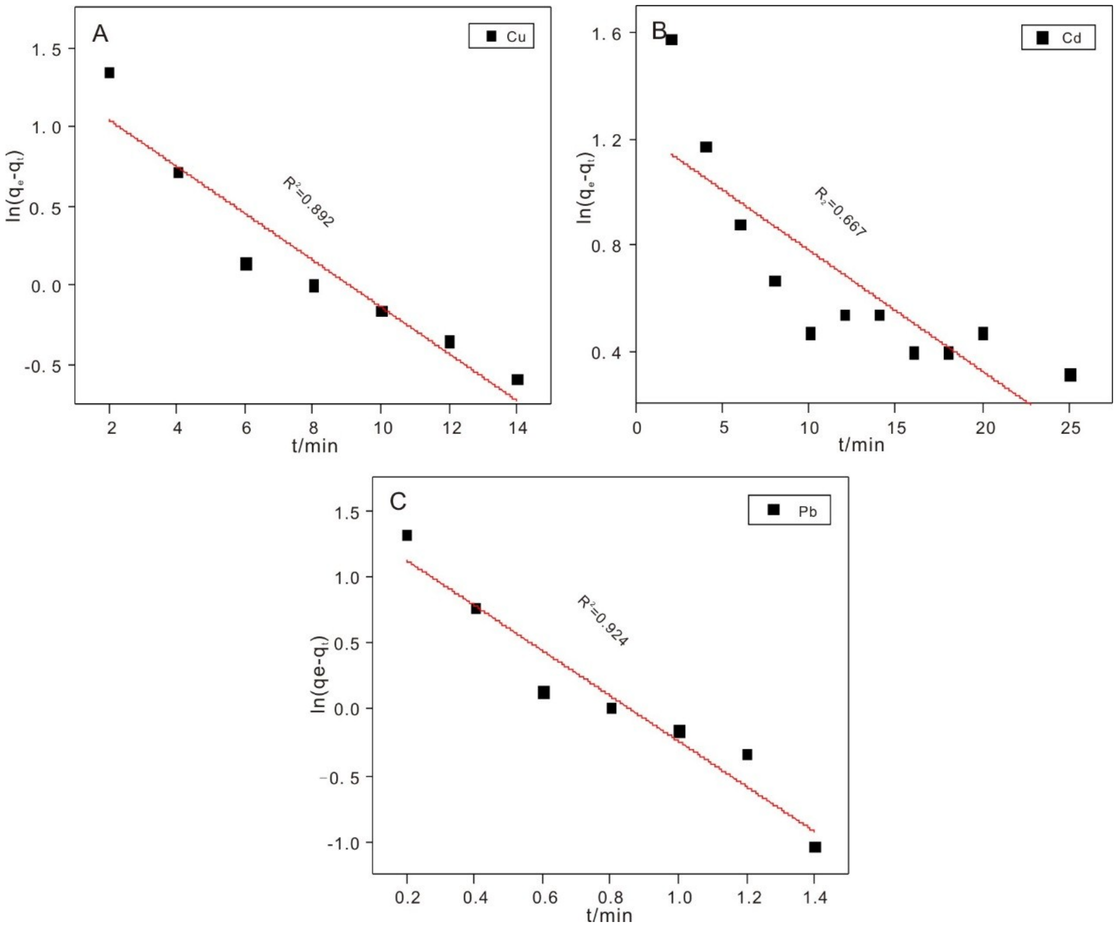

Fig. S2. The pseudo-first-order kinetic plots for the adsorption of (A) Cu^{2+}, (B) Cd^{2+}

and (C) Pb^{2+} on the surface of the NTA-modified silica gel. (Adsorbent dose:1.0 g/L,25 °C, pH: 5.0)

S3. Adsorption isotherm models for Cu^{2+}, Cd^{2+} and Pb^{2+} on NTA-modified silica gel

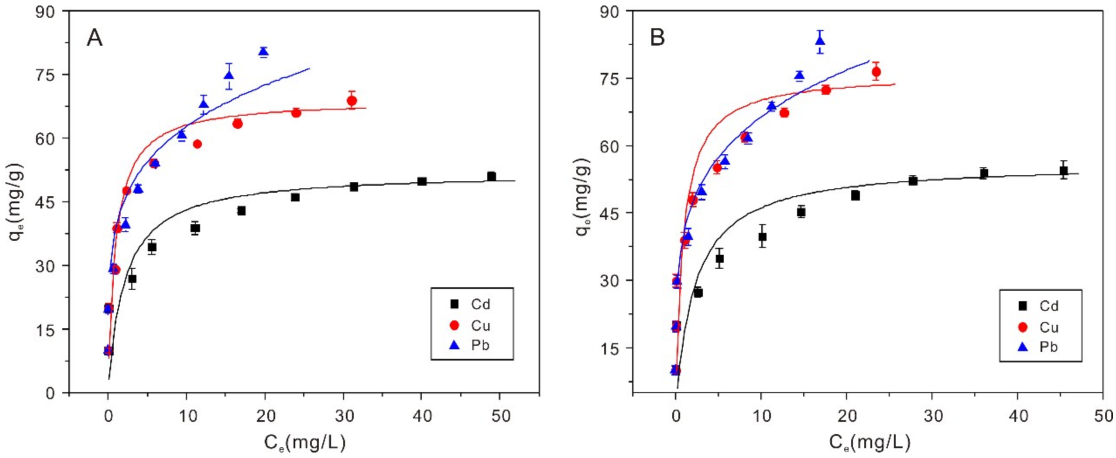

Fig. S3. Cu^{2+}, Cd^{2+} and Pb^{2+} ions adsorption isotherms onto NTA-modified silica gel at (A) 313 K and (B) 323 K (Adsorbent dose: 1.0 g/L, pH: 5.0).

S4. Langmuir and Freundlich isotherm models for fitting of Cu^{2+}, Cd^{2+} and Pb^{2+} on NTA-silica gel

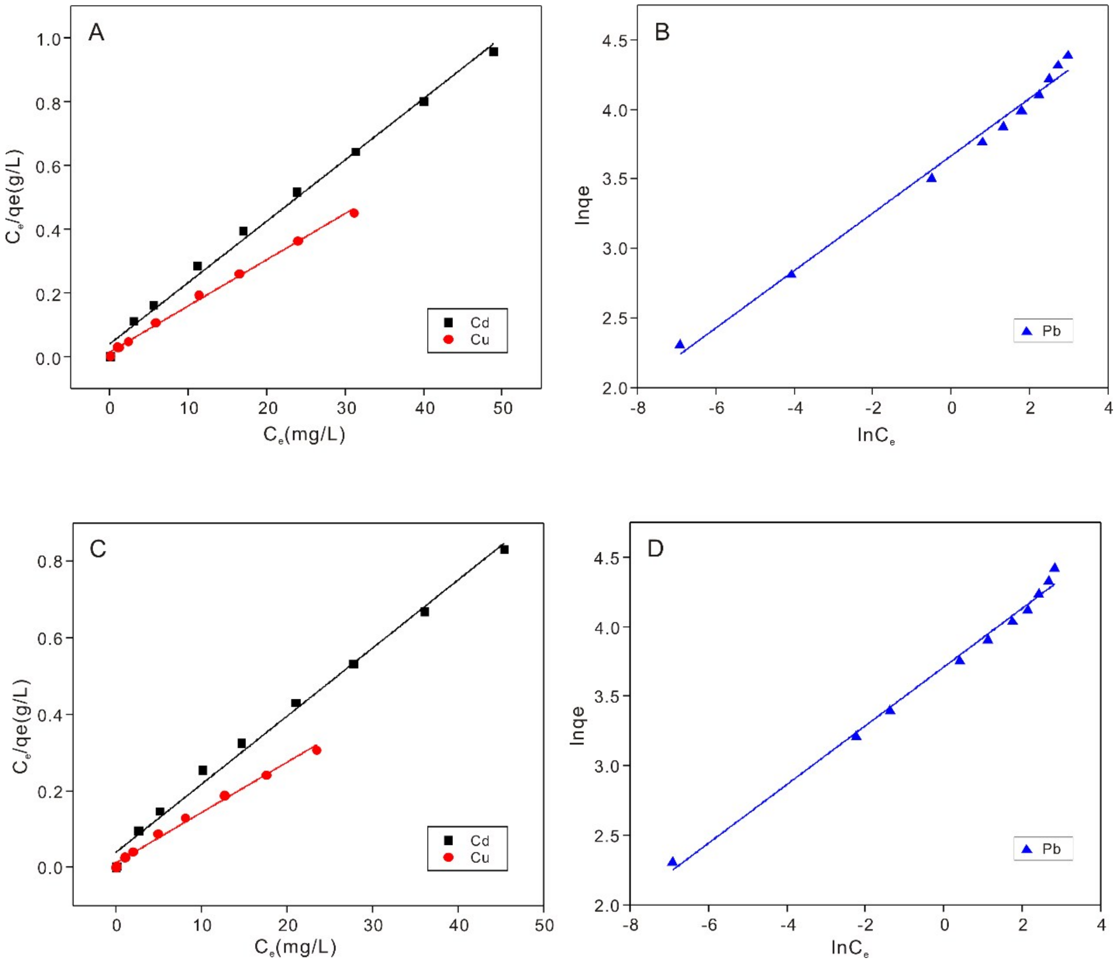

Fig. S4. Langmuir adsorption models for fitting of Cu^{2+} and Cd^{2+} at (A) 313 K and (C) 323 K and Freundlich adsorption models for fitting of Pb^{2+} at (B) 313 K and (D) 323 K on NTA-silica gel.

S5. Ill-suited adsorption isotherm models for Cu^{2+}, Cd^{2+} and Pb^{2+} on NTA-modified silica gel

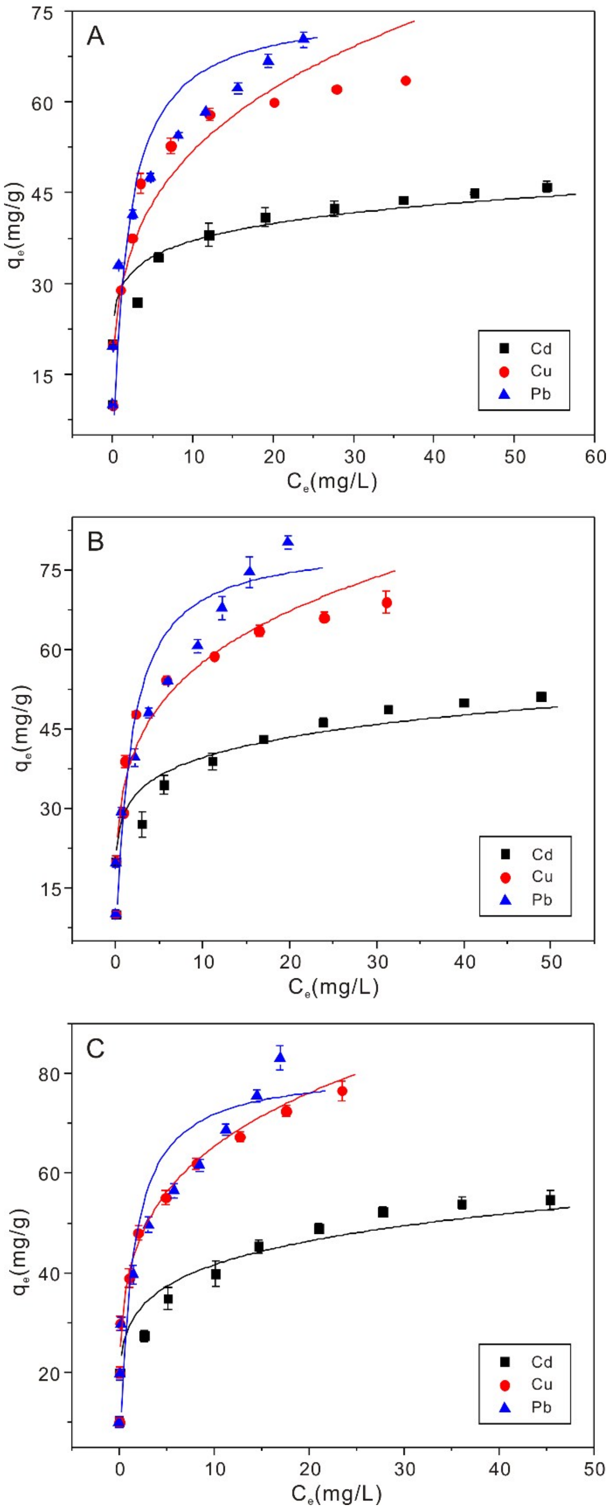

Fig. S5. Cu^{2+}, Cd^{2+} and Pb^{2+} ions adsorption isotherms onto NTA-modified silica gel at (A) 298 K, (B) 313 K and (C) 323 K (Adsorbent dose: 1.0 g/L, pH: 5.0).

S6. Langmuir and Freundlich isotherm models for fitting of Cu^{2+}, Cd^{2+} and Pb^{2+}

on NTA-silica gel

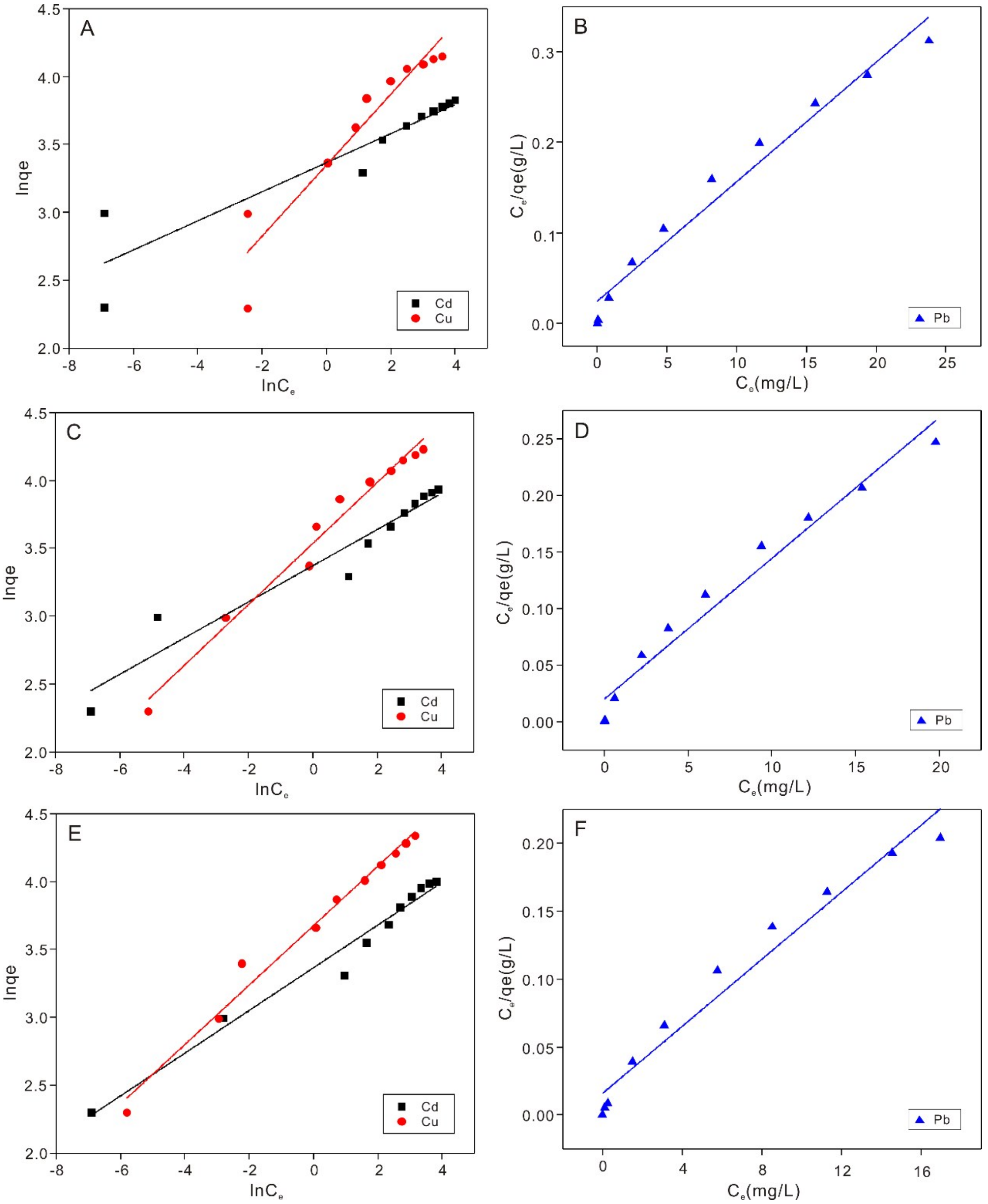

Fig. S6. Langmuir adsorption models for fitting of Cu^{2+} and Cd^{2+} at (A) 298 K, (C) 313 K and (E)323 K and Freundlich adsorption models for fitting of Pb^{2+} at (B) 298 K, (D) 313 K and (F) 323 K on the NTA-silica gel.

S7. Calculation of thermodynamic data

The $\ln K_d$ as function of C_e at T = 298 K, 308 K and 318 K was given in Fig. S7. The adsorption equilibrium constants (K^0) at different temperature were obtained by linear while C_e was equal to zero. At T = 298 K, 313 K, 323 K, $\ln K^0$ = 3.66, 4.40, 5.00, and $\triangle G^0$ = -9.06, -11.43, -13.44 kJ/mol, respectively for Cu^{2+}; $\ln K^0$ = 5.24, 4.83, 4.41 and $\triangle G^0$ = -12.99,-12.57, -11.85 kJ/mol, respectively for Cd^{2+}; $\ln K^0$ = 4.87, 5.26, 5.14 and $\triangle G^0$ = -12.08,-13.70, -13.80 kJ/mol, respectively for Pb^{2+}. The values of $\triangle H^0$ and $\triangle S^0$ can be calculated from the plot of $\ln K^0$ vs 1/T (Fig. S7). The value of $\triangle H^0$ and $\triangle S^0$ was calculated to be 42.67, -26.10, 24.05 kJ/mol and 173.38, -43.77, 121.22 J/mol K for Cu^{2+}, Cd^{2+}, and Pb^{2+}, respectively.

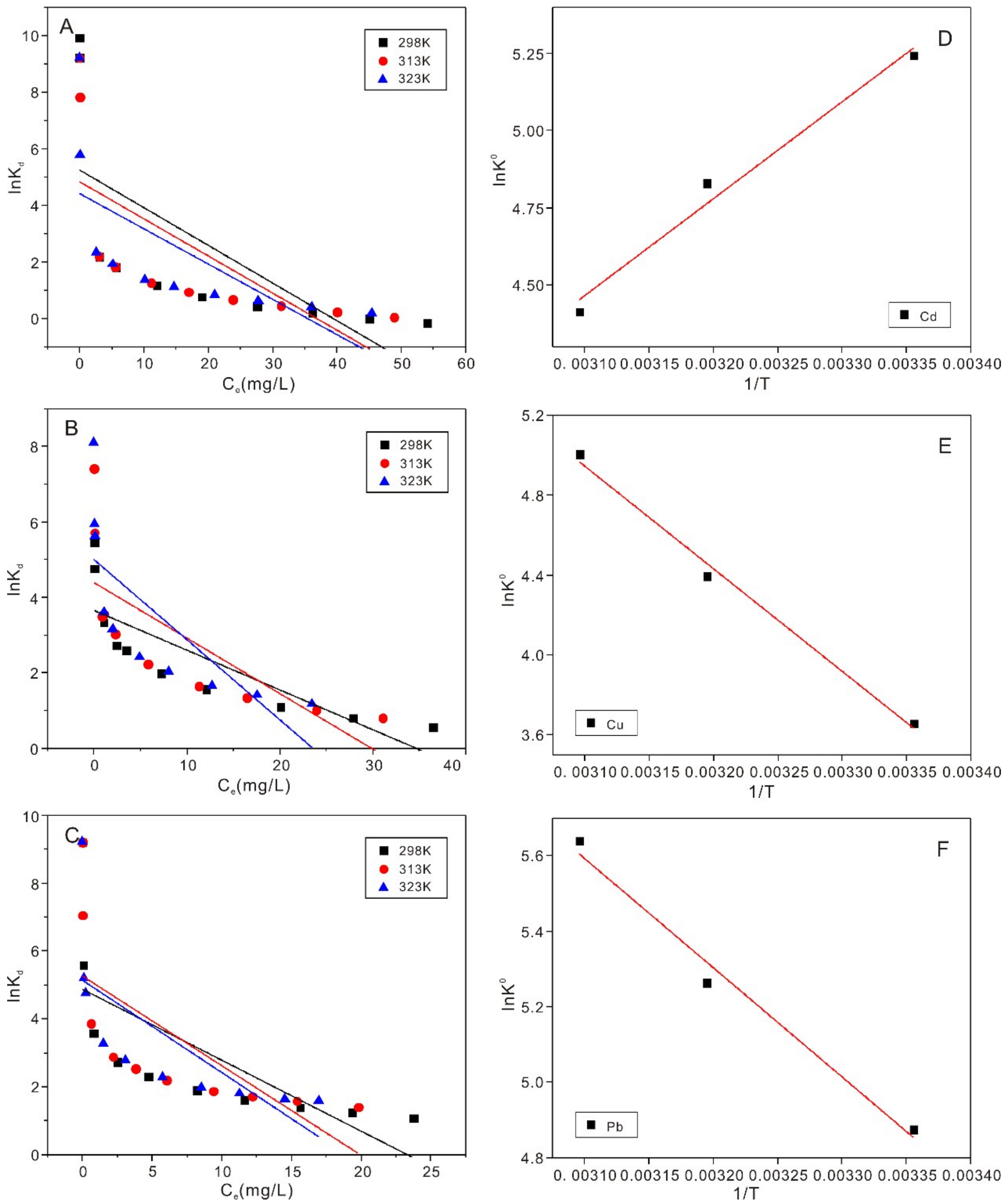

Fig. S7. (A), (B), (C) Linear plots of $\ln K_d$ vs C_e and (D), (E), (F) linear plot of $\ln K^0$ vs 1/T for the adsorption of Cu^{2+}, Cd^{2+} and Pb^{2+}on NTA-silica gel at 298, 308 and 318 K.

S8. Comparison about adsorption of non-and NTA-modified silica gel

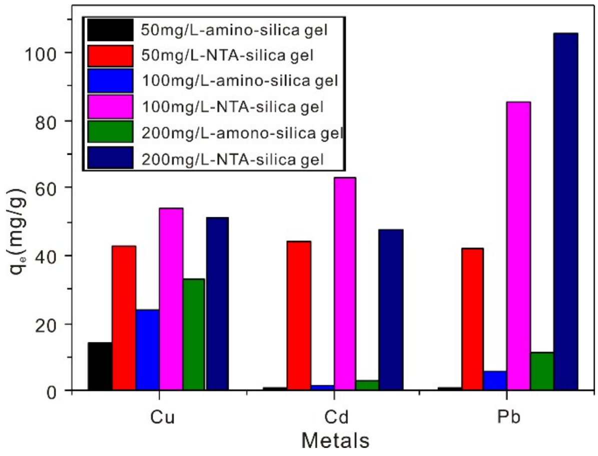

Fig. S8. Compare about adsorption of non- and NTA-modified silica gel with different concentrations (50, 100, 200 mg/L, adsorbent dose: 1.0 g/L, pH: 5.0)

S9. XPS spectra of NTA-silica gel before and after adsorption of the heavy metals

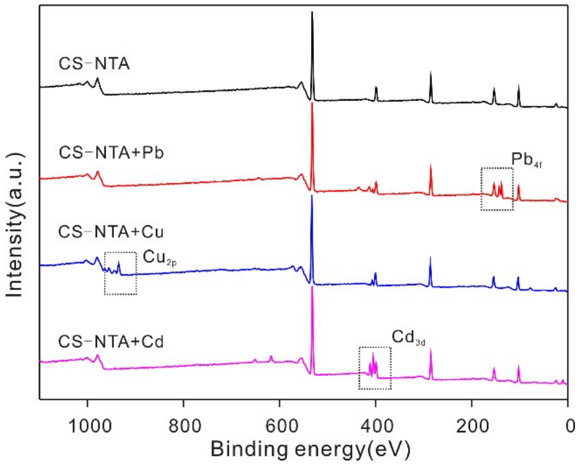

Fig. S9. XPS spectra of NTA-silica gel before and after adsorption of Cu^{2+}, Cd^{2+} and Pb^{2+}.

S10. FTIR spectra of NTA-silica gel before and after adsorbing heavy metals.

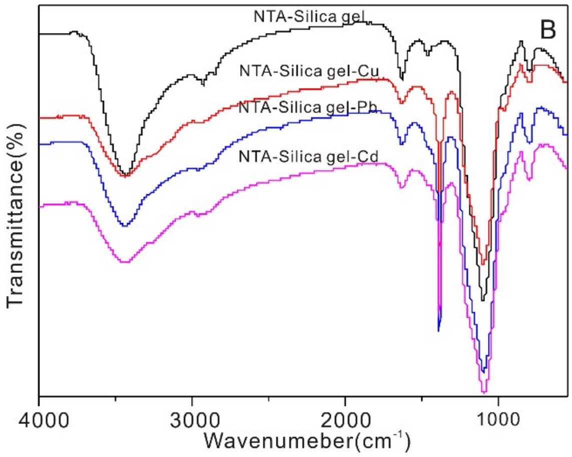

Fig. S10. FTIR spectra of NTA-silica gel before and after adsorbing Cu^{2+}, Cd^{2+} and Pb^{2+}.
